# Not only Alagille syndrome. Syndromic paucity of interlobular bile ducts secondary to HNF1β deficiency: a case report and literature review

**DOI:** 10.1186/s13052-019-0617-y

**Published:** 2019-02-21

**Authors:** Michele Pinon, Michele Carboni, Davide Colavito, Fabio Cisarò, Licia Peruzzi, Antonio Pizzol, Giulia Calosso, Ezio David, Pier Luigi Calvo

**Affiliations:** 1 Pediatric Gastroenterology Unit, Regina Margherita Children’s Hospital, Azienda Ospedaliero-Universitaria Città della Salute e della Scienza, University of Turin, Piazza Polonia 94, 10126 Turin, Italy; 2 Postgraduation School of Pediatrics, Regina Margherita Children’s Hospital, Azienda Ospedaliero-Universitaria Città della Salute e della Scienza, University of Turin, Turin, Italy; 3 Research & Innovation (R&I Genetics) srl, Padua, Italy; 4 Pediatric Nephrology Unit, Regina Margherita Children’s Hospital, Azienda Ospedaliero-Universitaria Città della Salute e della Scienza, University of Turin, Turin, Italy; 5 Department of Pathology, Azienda Ospedaliero-Universitaria Città della Salute e della Scienza, University of Turin, Turin, Italy

**Keywords:** Paucity of interlobular bile ducts, HNF1β mutations, Alagille syndrome, Ciliopathy, Renal cysts

## Abstract

**Background:**

paucity of interlobular bile ducts is an important observation at liver biopsy in the diagnostic work-up of neonatal cholestasis. To date, other than in the Alagille syndrome, syndromic paucity of interlobular bile ducts has been documented in four cholestatic neonates with HFN1β mutations. A syndromic phenotype, known as renal cysts and diabetes syndrome (RCAD), has been identified. This is usually characterized by a wide clinical spectrum, including renal cysts, maturity-onset diabetes of the young, exocrine pancreatic insufficiency, urogenital abnormalities and a not well established liver involvement. Herein we report a novel case of paucity of interlobular bile ducts due to an HFN1β defect.

**Case presentation:**

A 5-week-old boy was admitted to our department for cholestatic jaundice with increased gamma-glutamyl transpeptidase and an unremarkable clinical examination. He had been delivered by Caesarian section at 38 weeks’ gestation from unrelated parents, with a birth weight of 2600 g (3rd percentile). Screening for cholestatic diseases, including Alagille syndrome, was negative except for a minor pulmonary artery stenosis at echocardiography and a doubt of a thoracic butterfly hemivertebra. The finding of hyperechogenic kidneys with multiple bilateral cortical cysts at ultrasound examination, associated with moderately impaired renal function with proteinuria, polyuria and metabolic acidosis, was suggestive of ciliopathy. A liver biopsy was performed revealing paucity of interlobular bile ducts, thus the diagnosis of Alagille syndrome was reconsidered. Although genetic tests for liver cholestatic diseases were performed with negative results for Alagille syndrome (JAG1 and NOTCH2), a *de-novo* missense mutation of HNF1β gene was detected. At 18 months of age our patient has persistent cholestasis and his itching is not under satisfactory control.

**Conclusions:**

Alagille syndrome may not be the only syndrome determining paucity of interlobular bile ducts in neonates presenting with cholestasis and renal impairment, especially in small for gestational age newborns. We suggest that HNF1β deficiency should also be ruled out, taking into consideration HNF1β mutations, together with Alagille syndrome, in next generation sequencing strategies in neonates with cholestasis, renal impairment and/or paucity of interlobular bile ducts at liver biopsy.

## Background

Neonatal cholestasis is characterized by conjugated hyperbilirubinemia and manifests clinically with jaundice, pruritus, failure to thrive, fat-soluble vitamin deficiency and xanthomas. There may also be hypocholic or acholic stools in the presence of functional or anatomic biliary obstruction. Diagnostic work-up is of paramount importance to exclude biliary atresia, as the timing of surgical intervention directly impacts clinical outcomes.

Liver biopsy adds essential information to the diagnostic evaluation in persistent neonatal cholestasis, prompting clinicians to consider biliary atresia if ductular proliferation is present. As recently reported, paucity of interlobular bile ducts (PILBD) is not such a rare finding at histology, especially in infants with cholestasis (70/632 of pediatric liver biopsies, not considering graft versus host disease, drugs, chronic rejection) [1]. Cholestasis due to PILBD is caused by an alteration in the anatomic integrity of the biliary tract with absence of, or a marked decrease in, the number of interlobular bile ducts. PILBD can be only documented histologically as a loss of intrahepatic bile ducts in more than 50% of the portal tracts in a biopsy specimen containing at least 10 portal tracts. Two PILBD categories have been recognized: syndromic (S-PILBD) and nonsyndromic (NS-PILBD). S-PILBD is associated to Alagille syndrome (AGS) and is variably characterized by the presence of at least three of the five following features: PILBD-associated chronic cholestasis, peripheral pulmonary artery stenosis, vertebrae segmentation anomalies, characteristic facies and posterior embryotoxon. Moreover, renal and vascular alterations are often present in numerous AGS patients, even if they are not included in the diagnostic criteria. AGS is commonly associated with mutations in JAG1 gene, which encodes a protein involved in Notch signaling (AGS type 1), or in NOTCH2 gene (AGS type 2). NS-PILBD is a non-specific condition of unknown etiology and is not associated with systemic malformations or other disorders that induce biliary ductopenia. Despite medical treatment, end stage liver disease due to persistent cholestasis may occur in children with PILBD [2], as well as intractable pruritus, affecting the quality of life, failure to thrive and osteodystrophy [3]. Taken together (S-PILBD and NS-PILBD), liver transplantation is necessary in 30–40% of these patients and PILBD accounts for 5–10% of all the indications for pediatric liver transplantation [1, 3, 4]. That is why PILBD is an important finding in the diagnostic work-up of neonatal cholestasis, which should alert clinicians to consider a diagnosis of AGS, together with the result of echocardiogram, imaging of the vertebrae and ophthalmologic examination.

To date, to the best of our knowledge, only AGS has been associated with syndromic PILBD and there are few reports on NS-PILBD of unknown origin [5]. However, although no other underlying causes of syndromic PILBD have yet been well established, PILBD has been documented in a low number of cholestatic neonates with HNF1β mutations [6–10].

## Case presentation

A 5-week-old boy was admitted to our department for jaundice and failure to thrive. He had been delivered in another neonatal centre by Cesarean section, from nonconsanguineous healthy parents, at 38 weeks of gestation, with an Apgar score of 9/9. His birth weight was 2600 g (3rd percentile), length 49 cm (27th percentile) and cranial circumference 32.5 cm (5th percentile). Urinary Cytomegalovirus test was negative, as was his family history for known diabetes, hepatic or renal disease; he had a healthy 8-year-old brother. The baby had been discharged from the other centre, in a satisfactory condition, on the 4th day of life.

Our physical examination was unremarkable, except for skin and scleral jaundice. The baby also had hypocholic stools. Routine blood tests confirmed cholestatic jaundice (total bilirubin 11.95 mg/dL, conjugated bilirubin 6.69 mg/dL) with increased gamma-glutamyl transpeptidase (GGT 221 U/L), which persisted after ursodeoxycholic acid treatment (20 mg/Kg/day). Fat-soluble vitamins supplementation was started and cow’s milk with highly hydrolyzed proteins enriched with medium chain triglycerides was recommended.

Routine screening for cholestatic diseases, including primary investigations for Alagille syndrome, was negative except for a minor pulmonary artery stenosis at echocardiography and a doubt of a thoracic butterfly hemivertebra. Abdominal ultrasound (US) examination revealed a normal liver for size and echogenicity, normal biliary intrahepatic and extrahepatic tree, regular liver vessel flow and hyperechogenic kidneys, with multiple bilateral cortical cysts (maximum size 2 mm). Renal function was impaired (serum creatinine 0.59 mg/dL, estimated glomerular filtration rate 35 ml/min/1.73m^2^, Chronic Kidney Disease KDIGO stage 3), with metabolic acidosis and tubular proteinuria; he also had polyuria (7 mL/Kg/h) during hospitalization with depressed bregmatic fontanelle. The laboratory tests performed during hospitalization and follow-up are reported in Table 1.

**Table 1:** Laboratory tests

**Tests**	Reference range	5 weeks	2 months	6.5 months	9 months	11 months	14 months	16 months	18 months
Liver function tests									
Bilirubin total (mg/dL)	< 1	11.95	15.5	4.3	1.6	1.3	2.2	2.5	2.8
Conjugated bilirubin (mg/dL)	< 0.2	6.68	13.5	4.1	1.5	1.2	2.1	2.2	2.4
AST (IU/L)	< 50	210	276	86	75	92	189	135	87
ALT (IU/L)	< 40	320	349	47	63	90	332	177	77
GGT (IU/L)	< 50	221	1631	986	872	1129	1629	969	657
bile acids (mg/dL)	< 10	–	150	264	–	117	304	253	132
Albumin (g/dL)	3.6–5.2	–	3.7	3.3	3.7	4	4	4.2	4.2
Cholesterol (mg/dL)	< 200	–	361	–	270	–	349	256	–
HDL cholesterol (mg/dL)	> 40	-	-	-	-	-	133	101	-
LDL cholesterol (mg/dL)	< 90	-	-	-	-	-	179	131	-
Triglycerides (mg/dL)	< 105	–	–	135	138	119	187	120	78
Kidney function tests									
Serum creatinine (mg/dL)	0.18–0.33	0.58	–	0.5	0.51	0.5	0.47	0.46	0.50
eGFR* (ml/min/1.73m^2^)		37	–	55	56	62	66	67	66
Uric acid (mg/dL)	< 6	–	–	–	3.6	3.8	3.2	2.5	2.8
PrU/CrU** (mg/mg)	< 0.2	1.7	–	0	0	0	0	0.2	0
Hemoglobin (g/dL)	10.5–13.5	–	9	10.7	9.9	13.6	11.1	11.6	10.8

*estimated glomerular filtration rate (Schwartz’s eGFR = 0.413 x length/sCr)

**proteinuria/creatininuria index

**Fig. 1:**
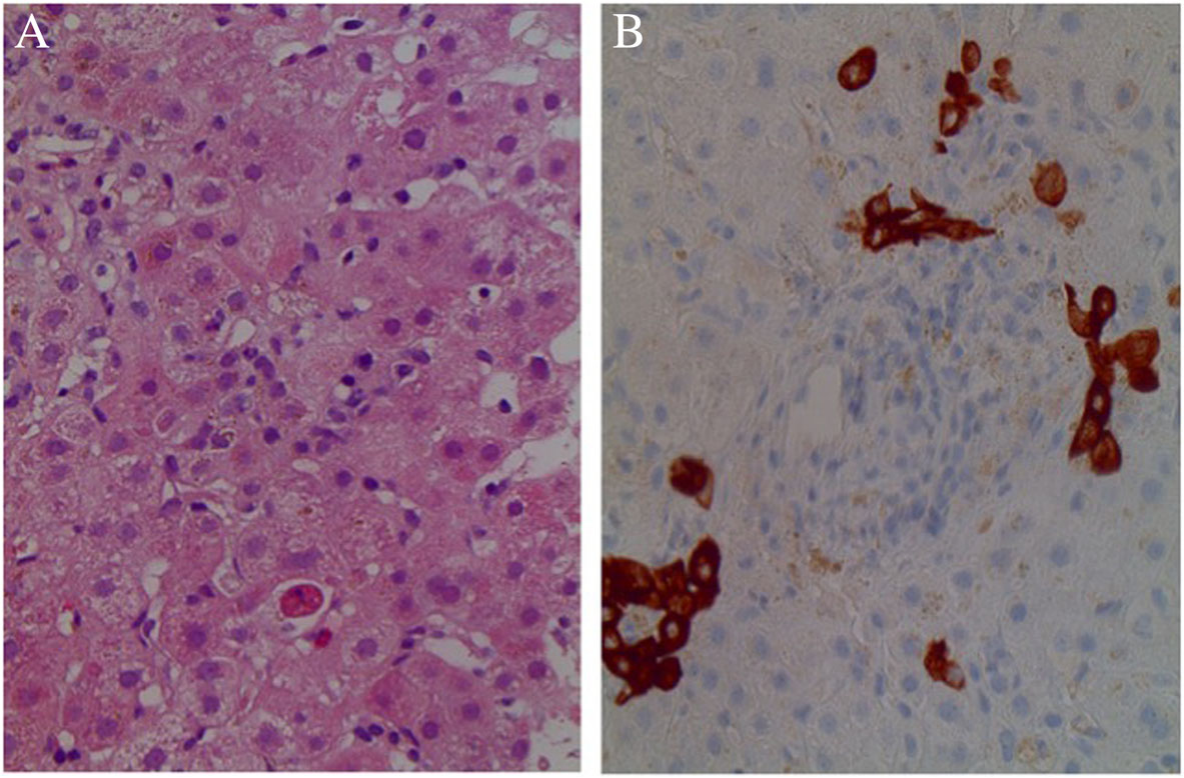
Histology of liver biopsy **a** Paucity of intrahepatic bile ducts with mild Kupffer cell activation, mild hepatocitic polymorphism, focal eosinophilic degeneration with a Councilman body; compatible with lobular light hepatitis. H&E 250X. **b** Interlobular portal tract with a ductular reaction resembling a ductal plate malformation. Cytokeratin 7250X.

The presence of hyperechogenic kidneys, with multiple bilateral cortical cysts at US examination, associated with a moderate alteration of renal function, were suggestive of ciliopathy. Hepato-biliary scintigraphy showed no passage of bile. A liver biopsy was performed, revealing PILBD with biliary stasis (Fig. 1). The association of cholestasis and PILBD, other than the renal involvement, led us to reconsider the diagnosis of AGS and to perform genetic tests for liver cholestatic diseases.

The baby continued to have hypocholic stools and persistent cholestasis whilst hospitalized. Although renal function was stable, a central venous access device was necessary for the first few weeks to treat the dehydration caused by polyuria. Moreover, a mild hyperparathyroidism was documented and subcutaneous erythropoietin (EPO) administration was started to treat a progressive anemia.

He was discharged from our department at the age of 2 months. At the first monthly follow-up, renal function and proteinuria were stable. Because of the persistence of hyponatremia and metabolic acidosis with hyperkalemia, supplemental oral rehydration with sodium chloride and administration of sodium bicarbonate were continued. EPO treatment was still necessary to maintain normal hemoglobin levels. During the following months, his cholestasis remained stable, although the onset of itching required rifampicin administration. His bilirubin level then decreased significantly, reaching a plateau (total bilirubin 2.5 mg/dL, conjugated 2.2 mg/dL) and the color of his stools normalized. There were no alterations in hepatic synthesis indexes or alpha-fetoprotein levels and no signs of portal hypertension. Renal function had a slight improvement, then stabilized to mild chronic renal failure (Chronic Kidney Disease KDIGO stage 2). US examination revealed an enlarged liver with a slightly inhomogeneous structure, but no focal liver lesions. It also revealed bilateral hyperechogenic kidneys of reduced size, whereas cysts were no longer documented. A reduction of fecal pancreatic elastase, with an increase in fecal fat excretion, was observed, probably due to an initial pancreatic exocrine dysfunction. As no pancreatic hypoplasia was evidenced at US examination, a magnetic resonance cholangiopancreatography (MRCP) has been programmed.

Genetic tests for liver cholestatic diseases revealed negative results for AGS (JAG1 and NOTCH2). However, subsequent whole exome sequencing (WES) analysis and interpretation, together with variant prioritization analysis, highlighted the presence of a previously described [11–13] missense heterozygous mutation in the HNF1β gene, p.Arg276Gln (c.827G > A). The mutation is located in exon 4 of the HNF1β gene within the DNA binding domain, leading to the substitution of glutamine by arginine at codon 276 (R276Q). As this novel mutation was absent in the proband’s parents, we concluded that the patient was a carrier of a *de-novo* mutation. To the best of our knowledge, this is the first patient reported to be a carrier of the p.Arg276Gln mutation presenting with renal involvement associated with early onset cholestasis.

At time of writing the baby is 18 months of age, with persistent cholestasis and pruritus, and a normal neurological development.

## Discussion and conclusions

To date, neonatal cholestasis has been identified in 5 subjects with pathogenic HNF1β mutations, in most cases *de-novo* deletions [6–10]; to the best of our knowledge, our case is the 6th one. Liver biopsy documented the presence of PILBD, histologically similar to AGS, in 5/6 patients including our case [6–9]. This datum is currently lacking in the remaining case [10].

The hepatocyte nuclear factor 1β (HNF1β), also known as transcription factor 2 (TCF2), is a key regulator of organogenesis for organs derived from the ventral endoderm [14] and it is involved in transcriptional and functional regulation of the liver, kidneys, urogenital tract and pancreas [15]. To date, more than 50 heterozygous mutations in the HNF1β gene (17q12) have been described in adults and young children, including missense mutations, small insertions-deletions or whole-gene deletions [15]. HNF1β-related disorders are inherited with an autosomal dominant pattern, even if most of the mutations are reported to be *de-novo* (as high as 50% of cases) [15]. HNF1β mutations were first recognized in a small group of patients with maturity-onset diabetes of the young, defined as MODY type 5, a monogenic form of early-onset diabetes mellitus with onset before the age of 25 [16]. Approximately 50% of the patients with HNF1B mutations go on to develop diabetes, most likely as a result of impaired insulin secretion due to pancreatic hypoplasia, together with insulin resistance. The co-occurrence of non-diabetic renal disease in these patients led to the discovery of the importance of renal involvement in the presence of an HNF1β defect [17], probably one of the most commonly known monogenic causes of developmental renal disease [15]. Cystic disease is the predominant renal HNF1β-associated phenotype, characterized by cortical small cysts, usually noted after birth, even if enlarged bilateral hyperechogenic kidneys may be observed by prenatal ultrasonography. Clinical presentation varies a great deal and can range from normal or mild alteration of renal function to chronic kidney failure, up to end-stage renal disease, dialysis or renal transplantation [15]. Electrolyte abnormalities, such as hypomagnesemia [18] and hyperuricemia [19] with early-onset gout, may also be present. The syndrome associated with HNF1β defects is termed Renal Cysts and Diabetes Syndrome (RCAD, OMIM #137920), even if it is characterized by a larger clinical spectrum, which also includes pancreatic hypoplasia with exocrine insufficiency [20], urogenital abnormalities [21] and a neurological involvement with autism spectrum disorders and cognitive impairment [22]. Liver involvement is frequently reported as an asymptomatic rise in the levels of transaminases. Less frequently, it has been described as a cholestatic liver impairment, such as neonatal or late-onset cholestasis [7]. The patients presenting with neonatal cholestasis had similar histological results, showing PILBD associated to marked cholestasis and a variable degree of periportal fibrosis [6–10], as shown in Table 2. They were small for the gestational age (SGA) with a history of intrauterine growth restriction (IUGR), in contrast with most cholestatic neonates. Moreover, they had renal cysts or renal hyperechogenicity, two of them also had more complex renal malformations, such as unilateral kidney agenesis and renal dysplasia, with a variable degree of chronic renal insufficiency not requiring dialysis or renal transplantation. There was a long follow-up (> 10 years) in 60% of cases. Diabetes requiring insulin therapy occurred at an average age of 10 years in 3/5 cases and 2/5 had pancreatic hypoplasia with impaired pancreatic exocrine function. Urogenital malformations were present in only 1/5 cases and a mild cognitive impairment was observed in 2/5. Although there is no data on a genotype-phenotype correlation, noteworthy is the fact that the patient reported by *Raile* et al with whole gene deletion had the most serious phenotype [6]. A progressive resolution of cholestasis within the first year of life was observed after conservative therapy in 3/5 cases, with a persistent mild alteration of transaminases at follow-up. The 4th patient underwent Kasai portoenterostomy at 32 days of age as extrahepatic bile ducts were not visualized at explorative surgery, with a consequent reduction of liver function tests to mildly elevated values. The 5th patient had a completely different clinical course, in as much as there was a diagnosis of hepatocellular carcinoma with elevated alpha-fetoprotein levels at 16 months of age. He was transplanted and the histological evaluation of the explanted liver showed micronodular cirrhosis [10].

**Table 2:** Patients’ characteristics of subjects with HNF1β mutations presenting with neonatal cholestasis

Sex/origin GW/BW g (DS)	Liver involvement	Liver biopsy	Renal function and ultrasound	Pancreatic involvement	Growth	Urogenital malformations/cognitive impairement	HNF1β mutation	Follow-up duration	Reference
♂/Japan 39/2390 (−2.26)	- neonatal cholestasis, acholic stools - no abnormality of extrahepatic bile ducts at explorative surgery - cholestasis resolution at 9-month follow-up with a persistent mild transaminases alteration - transient hypercholesterolemia	PILBD, marked cholestasis	- multiple bilateral cysts (right, four 1–2 cm diameter cysts, left, one 1 cm diameter cyst) - mild chronic renal insufficiency	diabetes requiring insulin therapy at 13 years of age (polyuria and polydipsia, mild metabolic acidosis)	NA	absent/ mild	c.457C > A, p.H135N (missense mutation in exon 2, de novo or paternal: history of liver dysfunction and renal insufficiency in his paternal family)	13 years	Kitanaka S 2004 [9]
♂/Belgium (Sardinian origin) 37/1520 (−3.46)	- neonatal cholestasis, slightly enlarged liver - cholestasis resolution at 1- year follow-up with a persistent mild transaminases alteration - 3 episodes of cholangitis - high triglyceridemia (300 mg/dL)	PILBD, severe biliary stasis, slight periportal fibrosis	- left kidney agenesis, enlarged and hyperechogenic right kidney, multiple cortical cysts - progressive chronic renal insufficiency from 19 months	- diabetes requiring insulin therapy at 5 years of age without ketoacidosis - pancreatic atrophy with progressive exocrine pancreatic deficiency requiring enzyme substitution from the age of 16 years	final height of 162.1 cm (− 1.86 SD), BMI 19.0 Kg/m^2^ (− 0.62 SD)	absent/NA	499_504 delGCTCTG insCCCCT, A167FS (combination of a deletion and insertion in exon 2, de novo)	18 years	Beckers D 2007 [8]
♂/Germany 35/1780 (− 1.69)	- neonatal cholestasis, acholic stools - cholestasis resolution at 1 year follow-up with a persistent mild transaminases alteration - hypercholesterolemia (292 mg/dL) and hypertriglyceridemia (307 mg/dL)	PILBD	- severe malformations of both kidneys (cystic kidney dysplasia and hydronephrosis due to urethral stenosis) - chronic renal insufficiency	- diabetes requiring insulin therapy at 13 years of age - pancreatic hypoplasia with progressive exocrine pancreatic deficiency	final height of 133.9 cm (− 6.7 SD), BMI 17.3 Kg/m^2^ (− 2.1 SD)	inguinal hernia, abdominal testis/delayed psychomotor development	HNF1β deletion exons 1–9, de novo	18 years	Raile K 2009 [6]
♀/Czech Republic 38/2360 (− 1.60)	- neonatal cholestasis, acholic stools - Kasai portoenterostomy at 32 days of age as extrahepatic bile ducts were not visualized at explorative surgery - progressive increase in liver function tests, mainly cholestatic - multiple cysts in the left hepatic lobe (diameter from 2 to 7 mm)	PILBD, cholestasis without signs of bile duct proliferation	- multiple bilateral cortical cysts (maximal diameter 5 mm), prenatally hyperechogenic kidneys - normal renal function by 2-year follow-up - mild hypomagnesemia	- pancreatic hypoplasia (absent body and tail) without exocrine pancreatic deficiency - normoglycaemia by 2-year follow-up	growth along the 3rd centile	absent/absent	1698 kb deletion including HNF1β, de novo	2 years	Kotalova R 2015 [7]
♂/France 35/NA	- neonatal cholestasis without acholic stools - hepatocellular carcinoma with elevated alpha-fetoprotein levels at 16 months of age requiring liver transplant - no relapse at 1-year follow-up	- multinodular hepatic tumor and micronodular cirrhosis at the explant - no information available on PILBD	- renal hyperechogenicity - transient renal failure	NA	NA	NA/NA	1.5 Mb deletion including HNF1β	2 years	de Leusse C [10]
♂/Italy 38/2600 (− 1.27)	- neonatal cholestasis, hypocholic stools - persistent cholestasis and pruritus at 18-month follow-up - hypercholesterolemia (256 mg/dL) and hypertriglyceridemia (120 mg/dL)	PILBD, biliary stasis	- hyperechogenic kidneys, with multiple bilateral cortical cysts (maximum size 2 mm) - chronic renal insufficiency	initial pancreatic exocrine dysfunction without pancreatic hypoplasia at US	growth along the 10th centile	absent/absent	c.827G > A, p.R276Q (missense mutation in exon 4, de novo)	18 months	Present report

*NA*: Information not available, *GW*: Gestation weeks, *BW*: Birth weight, *PILBD*: Paucity of interlobular bile ducts, *BMI*: Body mass index, *US*: Ultrasound

Our patient’s clinical course was similar to 4/5 cases previously reported in literature [6–9], i.e. the presence of renal cysts with a moderate alteration of renal function and an incipient pancreatic insufficiency. Uric acid and magnesium levels were in the normal range, there were no urogenital malformations or evident neurological deficits. As would be expected, considering the short follow-up and young age of the patient, there was no diabetes at time of writing. Conversely, our patient differed from the other 3/5 cases as his cholestasis did not resolve within the first year of life, but it was stable at 18 months with persistence of poorly controlled itching, despite medical therapy.

The hepatic phenotype is consistent with the paucity of bile ducts observed in knock-out mice with a liver-targeted HNF1β deletion [23]. It has been reported that HNF1β could be necessary for intrahepatic bile duct morphogenesis during liver formation from the ductal plate, that is normally detected along the periportal mesenchyme during the embryonic period [23]. The inactivation of HNF1β in mice causes severe jaundice and growth retardation; histological analysis has demonstrated the persistence of the ductal plate after birth together with a strong decrease in intrahepatic bile ducts, most likely responsible for PILBD. Other abnormalities in mice include gallbladder and extrahepatic bile duct epithelial dysplasia and poor formation of interlobular arteries [23].

A biliary extrahepatic involvement has also been reported in humans, as in the patient with neonatal cholestasis and PILBD who underwent Kasai portoenterostomy [7]. Another report described biliary abnormalities, identified by MRCP, in six patients with HNF1B mutations. Most of them had varying types of bile duct cysts (BDCs) in the extrahepatic bile ducts, with an atypical morphology for any Todani classification [24].

All these alterations seem to have an underlying developmental origin from anomalies in ductal plate remodeling, resulting in ductal plate malformations (DPMs), characterized by the persistence of post-natal embryonic biliary structures, biliary cell clusters or duct-like structures [25]. HNF1β could well play a pivotal role as a regulator of primitive ductal structures (PDS). According to a new pathogenic classification, DPMs are not the result of a lack of PDS remodeling, but rather the common endpoint of different defects of differentiation, maturation, expansion, polarity and/or ciliogenesis of PDS, affecting distinct stages of bile duct morphogenesis: e.g. mice with HNF1β deficient livers showed a normal differentiation, but an abnormal PDS maturation [26].

These developmental anomalies represent the liver involvement in a wide variety of diseases that affect various organs, generally classified as ciliopathies [27]. The role of HNF1β in ciliogenesis has been evidenced by electron microscopy, demonstrating a reduction of normal primary cilia on the epithelia cells of cholangiocytes in liver biopsies from three adults with late-onset cholestasis and no structural intra- or extrahepatic bile duct defects [28]. That is why HNF1β is considered a ciliopathy gene included among the genetic defects of syndromic ciliopathies with liver involvement [29]. Abnormal biliary structures or bile duct cysts are a common finding in most ciliopathies [27]. However, in the presence of HNF1β defects, these abnormalities may involve a paucity or a complete lack of intrahepatic bile ducts. What we deduced from our case and other similar ones reported, is that a ciliopathy should also be considered when liver biopsy shows ductopenia, with negative investigations for AGS.

Next generation sequencing (NGS) strategies, such as WES or whole genome sequencing (WGS), are promising to discriminate neonatal monogenic cholestatic disorders and should play a pivotal role in the evaluation of cholestatic neonates in addition to liver biopsy results. In silico gene panel technology is another effective tool to perform targeted analysis of WES or WGS data [30]. We are of the opinion that it is advisable to take into consideration HNF1β gene mutations in WES or WGS data analysis, together with AGS gene defects, in neonates with cholestasis, renal impairment and/or PILBD at liver biopsy. Moreover, HNF1β gene should be included in NGS-expanded panels created for cholestatic disorders.

As for AGS, we also advocate an early genetic test in the presence of extrahepatic involvement to exclude a misdiagnosis of biliary atresia, avoiding the need for unnecessary explorative surgery [31].

The importance of clinical examination and timely follow-up must not be underestimated, as HNF1β mutations may lead to serious extrahepatic manifestations. Further studies in larger patient series are required so as to better define the prognosis of these patients, also considering the recent report of a cholestatic infant with hepatocellular carcinoma [10].

In conclusion, HNF1β deficiency is probably associated to a more prevalent and complex biliary phenotype than previously reported, with important clinical implications. HFN1β defects should be considered in neonates with cholestasis and renal impairment, especially in SGA and IUGR newborns with a family history of renal disease or diabetes. Ductopenia is an important finding in the diagnostic work-up of neonatal cholestasis that, however, requires thorough investigation to rule out causes other than AGS. Therefore, HNF1β deficiency should be taken into consideration as one of the underlying causes of S-PILBD, in addition to AGS.

Hopefully, our findings may add further information to the scarce documentation of this rare disease.

## Abbreviations

AGS: Alagille Syndrome
BDCs: bile duct cysts
DPMs: ductal plate malformations
EPO: erythropoietin
GGT: gamma-glutamyl transpeptidase
HNF1β: hepatocyte nuclear factor 1β
IUGR: intrauterine growth restriction
MODY5: maturity-onset diabetes of the young
MRCP: magnetic resonance cholangiopancreatography
NGS: next generation sequencing
PDS: primitive ductal structures
PILBD: paucity of interlobular bile ducts
RCAD: renal cysts and diabetes
SGA: small for the gestational age
TCF2: transcription factor 2
US: ultrasound
WES: whole exome sequencing
